# Screen-printed flexible carbon electrodes for efficient neural impulse transmission

**DOI:** 10.1038/s41598-025-27519-3

**Published:** 2025-12-11

**Authors:** Marek Pawlikowski, Zbigniew Gulbinowicz, Rafał Drobnicki, Daniel Janczak

**Affiliations:** Institute of Mechanics and Printing, Warsaw University of Technology, ul. Narbutta 85, 02-524 Warszawa, Poland

**Keywords:** Neural impulses, Carbon-based electrodes, Screen printing, Energy science and technology, Engineering, Materials science, Nanoscience and technology, Neuroscience

## Abstract

The paper presents research on the electrical performance of carbon-based electrodes printed by screen printing technique. The electrodes are studied with respect to their potential application as conductors of neural impulses in the treatment of neural system disorders. Three types of carbon are considered, i.e. graphite, carbon black, and graphene. The electrodes of various dimensions were printed on thin Mylar-A PET foil from Micel Sp. z o.o., Poland. The electrical performance of the electrodes is verified on a single rectangular electrical impulse. The ability of each electrode to conduct the input impulse is reported. Also, the electrical properties are measured and correlated with the conductive characteristics. The results demonstrate that graphite- and carbon-black-based electrodes provide superior electrical signal transmission, whereas graphene electrodes underperform. This finding suggests directions for future research on neural printable interfaces using carbon.

## Introduction

One of the most serious problems affecting human health is nervous system diseases. They lead to neurological disorders and neuropathies, which influence patients’ quality of life and present significant challenges for treatment and management. Problems with neural impulse transmission through the neurons may also have a traumatic origin. Usually, conservative treatment, like drug therapy or physiotherapy, is implemented in such cases. However, many studies have focused for years on artificial neural signal transmitters that would establish a specific link between impaired neurons and restore neural conduction excitations. Since those devices are electrical conductors, they will be referred to as electrodes in this paper^1^. It is worth noting that in the literature, the term “electrodes” in the context of neural signal transmission also encompasses devices used to monitor, detect, and record neural signals^2–4^.

Carbon and its allotropes appear to be the best materials for neural interfaces^5,6^. It is their chemical inertness, biocompatibility, good electrical performance, and electrochemical stability that make them superior to other materials for implantable neural electrodes. In addition, due to the channel nanostructure of carbon-based materials, their electrochemical performance can be improved^7^.

Carbon-based materials, such as graphene (GRN), graphite (GRT), and carbon black (CB), have been extensively used to fabricate electrodes for neural applications. Graphene is characterized by high conductivity, excellent flexibility, and biocompatibility, making it a promising material for stable electrode-nerve devices^8–10^. A sheet of graphene consists of a two-dimensional single-layer of sp^2^-hybrid carbon atoms hexagonally arranged^11^. It also has exceptional mechanical properties (Young modulus approx. 1 TPa^12^ and a large specific surface area (2630 m^2^ g^− 1^^13^. All those properties have made graphene a very promising material that can be utilized in many applications, including as a neural impulse transmitter.

Also, graphite has found broad application in biomedical areas due to its excellent properties, as well as its low cost and abundance in nature. It typically consists of more than a hundred stacked layers of graphene. This allotrope of carbon occurs naturally and is the most stable form of carbon under standard conditions. Graphite is a good conductor of heat and electricity and has excellent mechanical properties, i.e. high stiffness and strength^14^. It is a material with high chemical inertness and corrosion resistance. Natural graphite can be classified into three principal types^15^: crystalline small flake graphite (or flake graphite), lump graphite, and amorphous graphite (very fine flake graphite). Those types of GRT differ from each other in terms of physical properties, appearance, chemical composition, and impurities.

Carbon black is usually used as a filler in non-conductive materials to improve their mechanical and electrical properties. The specific surface area of CB and the degree of spherical primary particles aggregation are regarded as the two most important CB properties influencing the electrical conductivity of CB^16^. Its characteristics make it a material suitable for a neural electrode, which can be used to recover neural impulse transmission in impaired neurons.

Although carbon-based materials have been regarded as very good candidates for electrical conductors, other types of electrical materials have been developed in recent years, such as conductive polymers (CP). One of the most studied CPs is poly(3,4-ethylenedioxythiophene): polystyrene sulfonate (PEDOT: PSS). It demonstrates high transparency, good thermal stability, and excellent solution processing^17^. However, its practical application is hindered by structural degradation during ion intercalation/deintercalation cycles^18^. The charge-discharge process usually causes mechanical damage to a PEDOT: PSS electrode^19^. Nevertheless, some modifications have been applied to the polymer to enhance its performance^20–23^.

The present study demonstrates the application of three carbon-based materials in the fabrication of electrodes capable of transmitting nerve signals impulses. The research aims to elaborate and verify experimentally the composition of such electrodes. We adopted screen printing technique on thin foils to manufacture the neural devices. Generally, 3D printing technology has already been successfully used in carbon electrode fabrication^24^. The screen printing technique is highly advantageous because it enables the fabrication of electrodes of various shapes and compositions^25^. In our research, we considered GRT (fine-grained graphite MG1596 micro powder), GRN, and CB as the main components of the electrodes. However, other forms of carbon-based materials, such as carbon fiber, glassy carbon, or carbon nanotubes, are also used for electrode manufacturing.

## Materials and methods

### Electrode manufacturing

**Fig. 1:**
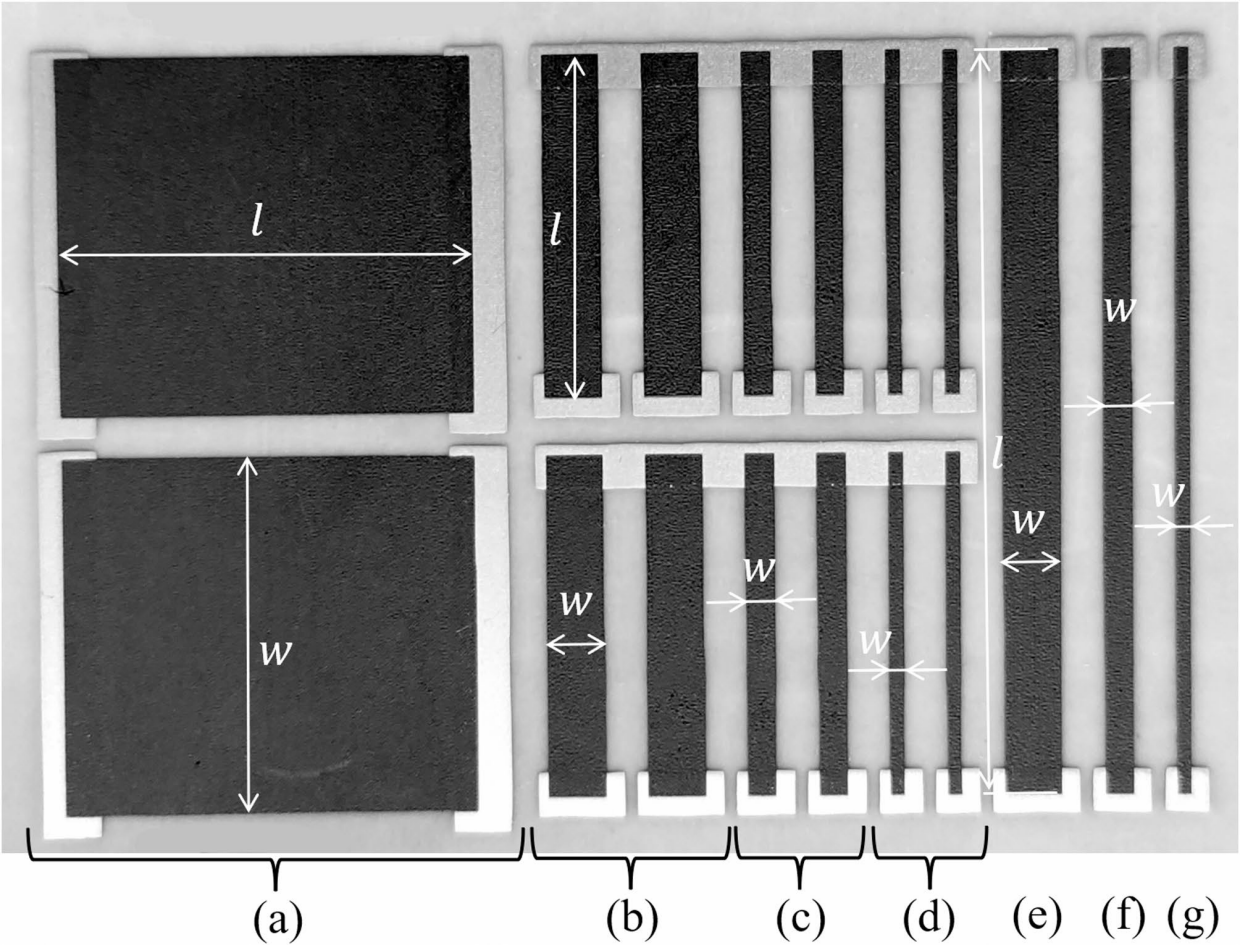
Dimensions of the printed MG1596, CB, and GRN electrodes ($\:w\:\text{x}\:l$): 25 × 30 mm (**a**), 4 × 25 mm (**b**), 2 × 25 mm (**c**), 1 × 25 mm (**d**), 4 × 52 mm (**e**), 2 × 52 mm (**f**), 1 × 52 mm (**g**).

The electrodes were produced using the screen printing technique on thin, biocompatible, flexible 0.125 mm thick foil made of Mylar-A PET from Micel Sp. z o.o Poland. The substrate passed cytotoxicity testing using the MTT assay according to PN-EN ISO 10993-5:2009 with the L929 cell line. The path conducting the impulses was made of a carbon-based paste. We have printed electrodes using paste with two types of binder, namely 24% weight vinyl chloride copolymer-based binder in the solvent 2-(2-Butoxyethoxy) ethyl acetate from Novelinks (LARO), and 8%.

polymethyl methacrylate PMMa MW350000 in the same solvent (PMMA). As the main component of the paste, we used fine-grained graphite MG1596 micro powder (10% and 30%), conductive carbon black CB (4% and 8%), and graphene GRN (10%). In paritcular, the MG1596 electrodes were printed with the 8 polymethyl methacrylate PMMa MW350000 in the solvent 2-(2-Butoxyethoxy) ethyl acetate from Novelinks, which will be referred to as PMMA, and the CB and GRN electrodes were printed with 24% weight vinyl chloride copolymer-based binder in the same solvent (2-(2-Butoxyethoxy) ethyl acetate from Novelinks), which will be referred to as LARO. The electrode labels used in the paper are shown in Table 1 along with comments. The thickness of all the printed electrodes was approx. 100 μm. The width $\:w$ ranged from 1 to 25 mm, while the electrode length $\:l$ was 25 mm, 30 mm, or 52 mm (Fig. 1). The same geometries were used for all the tested electrodes.

**Table 1:** Labels of the investigated electrodes.

Label	MG1596 30% PMMA	MG1596 10% PMMA	CB 8% LARO	CB 4% LARO	GRN 10% LARO
Comment	30% of fine-grained graphite MG1596 micropowder printed with PMMA	10% of fine-grained graphite MG1596 micropowder printed with PMMA	8% of carbon black CB printed with LARO	4% of carbon black CB printed with LARO	10% of graphene GRN printed with LARO

### Experimental setup

To determine the basic electrical characteristics of the electrodes, resistance and electrode response to the transmission of a rectangular signal were measured.

**Fig. 2:**
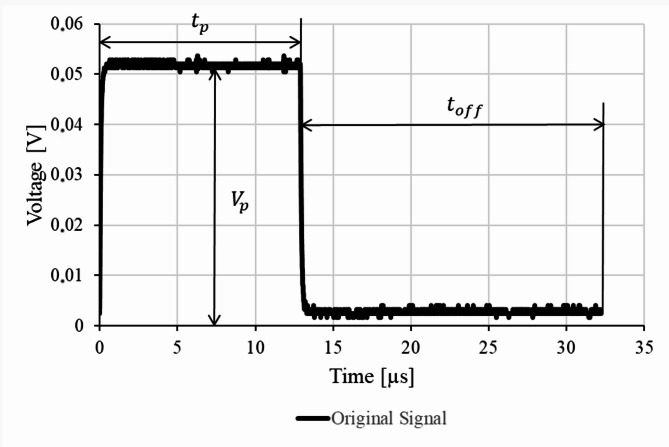
The parameters of the input signal used to verify the electrical performance of the tested electrodes.

**Fig. 3:**
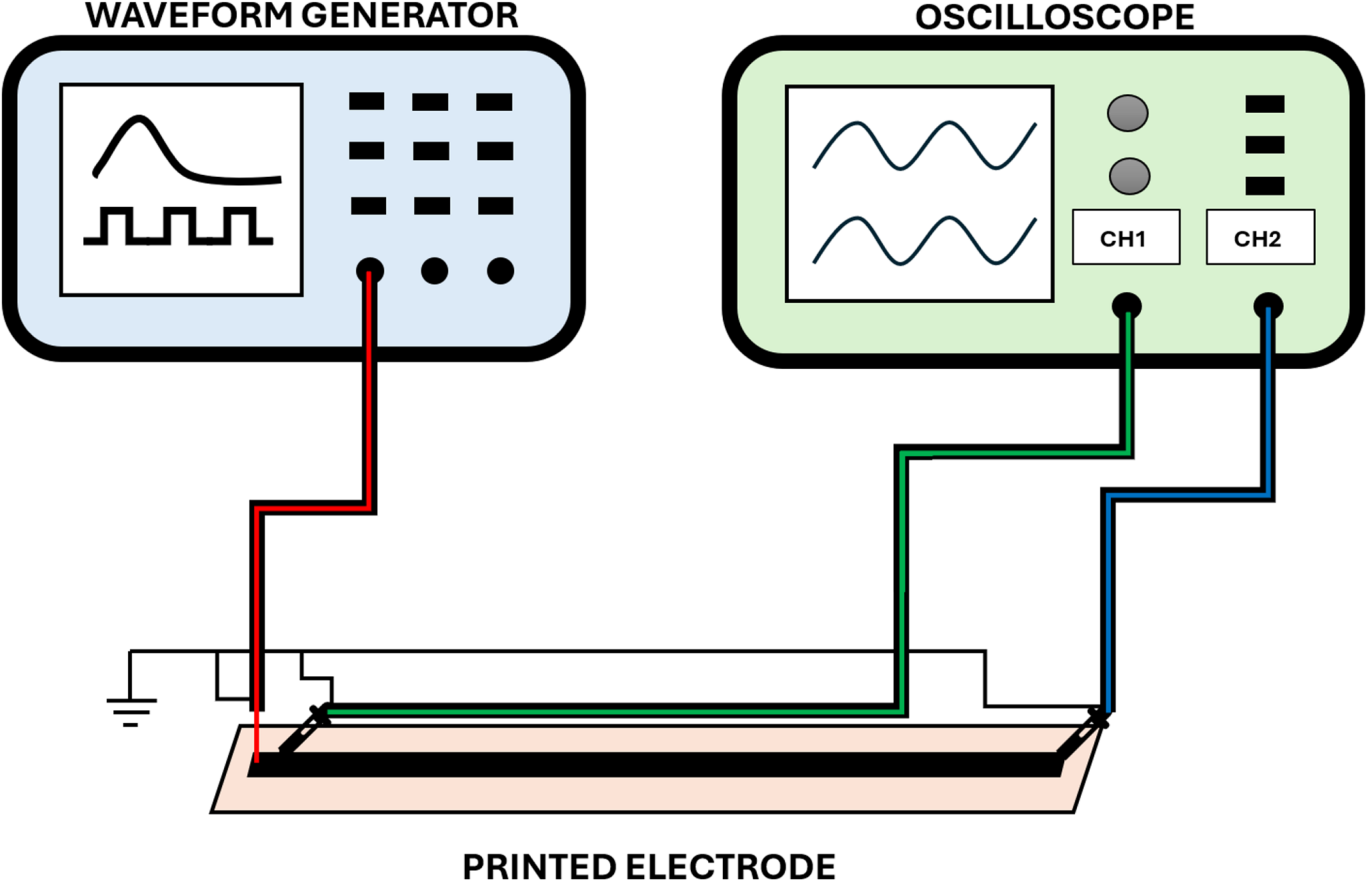
Experimental setup used in the electrical measurements.

During the experimental work, we subjected the electrodes to electrical impulses shown in Fig. 2, and compared the input signal with the output one from the electrodes. The impulse parameters were: $\:{V}_{p}=50$ mV, $\:{t}_{p}=13$ µs, $\:{t}_{off}=20$ µs. Although the time of neural impulses fluctuates from 1 ms to 100 ms or even more^26^, our electrical impulse time was altogether 0.033 ms. In our study, we verified the electrical performance of the printed electrodes based on a shorter impulse. Our intention was to study the electrical performance of the electrode under a high-frequency electrical signal. We hypothesize that such a procedure ensures proper electrode function in terms of their neural impulse conduction, which occurs at a lower frequency. The hypothesis is based on the assumption that the electrical tests did not affect the properties of the electrodes. Thus, their electrical performance remains the same. The measurements were conducted by means of a UTG1022X waveform generator and a SIGENT SDS1022CML+ oscilloscope. To ensure proper system measurement and system stability, the grounding of the circuit should be properly defined. In our studies, all the circuit branches were connected to a common ground. The experimental setup is schematically shown in Fig. 3. The voltage impulses were generated by the generator and applied to the electrodes. The oscilloscope gathered the voltage signal from the other end of the electrodes. The data of the two signals was saved in files to visualize the outputs. The impedance was measured by a standard electric meter.

## Results

**Fig. 4:**
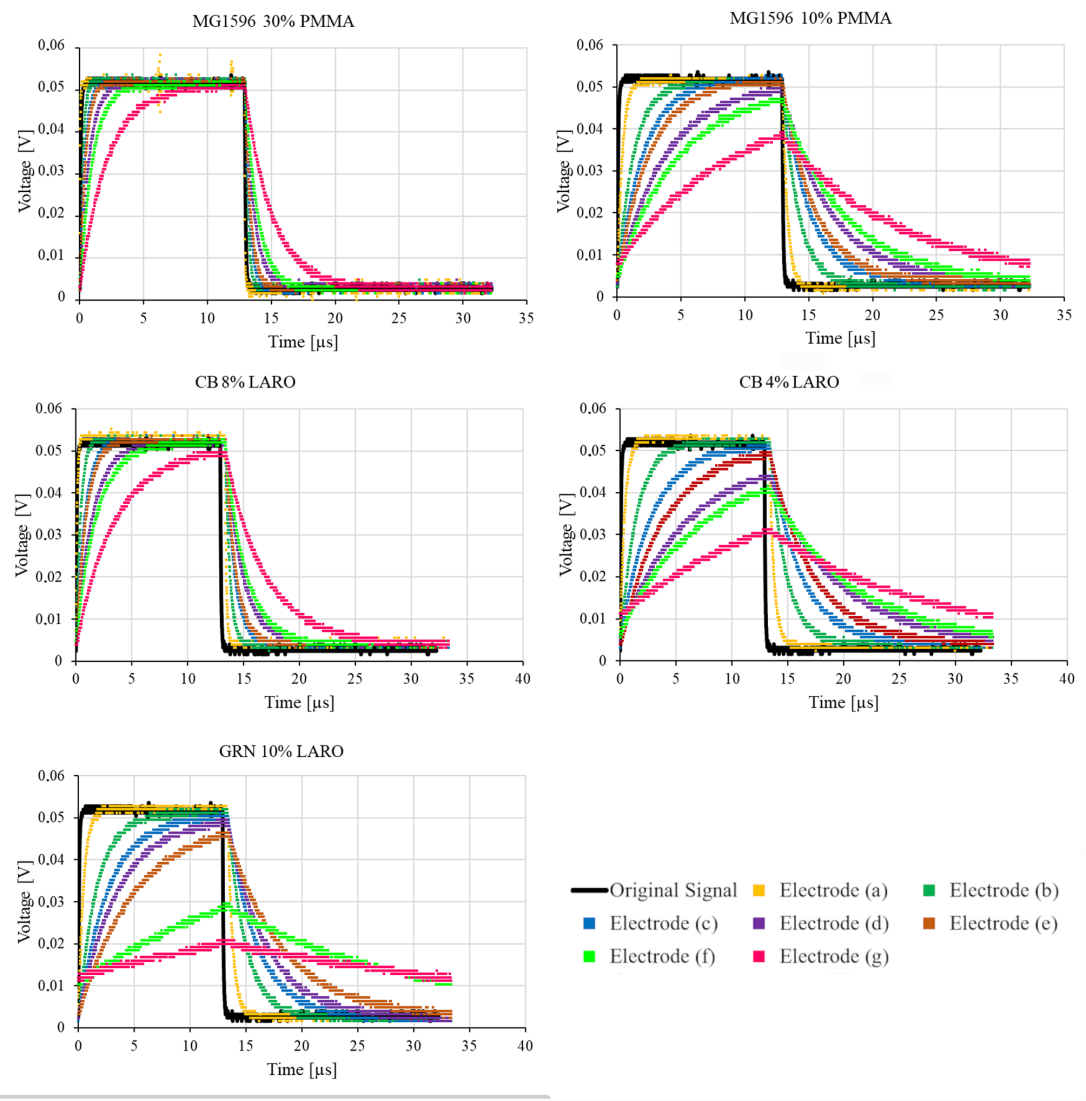
The output signals registered in the electrodes.

The results of the measurements are displayed graphically in Fig. 4, showing each electrode’s ability to conduct the original impulse. The quantitative performance of the studied electrodes is presented in Table 2 as correlation coefficient values.

**Table 2:** Correlation coefficient values for the electrodes ($\:\stackrel{-}{\sigma\:}$ denotes standard deviation).

	MG1596 30% PMMA	MG1596 10% PMMA	CB 8% LARO	CB 4% LARO	GRN 10% LARO
(a)	0.99765	0.977272	0.996887	0.977344	0.974463
(b)	0.99	0.927938	0.982299	0.911527	0.868775
(c)	0.978612	0.867731	0.958341	0.817623	0.822455
(d)	0.954974	0.754021	0.908587	0.606153	0.797025
(e)	0.973245	0.825369	0.948199	0.734571	0.65347
(f)	0.936227	0.658749	0.885309	0.524678	0.260416
(g)	0.854428	0.435365	0.763759	0.310569	0.195449
$\:\stackrel{-}{\sigma\:}$	0.049	0.18	0.08	0.23	0.31

The correlation coefficient represents numerically to what degree the signal passed through an electrode coincide the original signal.

**Table 3:** Values of resistive characteristics of the electrodes ($\:\stackrel{-}{\sigma\:}$ denotes standard deviation).

	MG1596 30% PMMA		MG1596 10% PMMA		CB 8% LARO		CB 4% LARO		GRN 10% LARO	
	$\:R$ [Ω]	ρ_*R*_ [Ω mm]	$\:R$ [Ω]	ρ_*R*_ [Ω mm]	$\:R$ [Ω]	ρ_*R*_ [Ω mm]	$\:R$ [Ω]	ρ_*R*_ [Ω mm]	$\:R$ [Ω]	ρ_*R*_ [Ω mm]
(a)	300	25	2700	225	808	67	3090	258	3130	261
(b)	1500	24	9000	144	3700	59	14,000	224	1400	22
(c)	2900	23	16,500	132	7700	62	29,000	232	22,000	176
(d)	6000	24	29,000	116	15,200	61	63,350	253	31,000	124
(e)	3500	27	20,800	160	9060	70	40,200	309	42,000	323
(f)	8400	32	42,100	162	18,570	71	81,100	312	162,000	623
(g)	18,100	35	83,600	161	36,800	67	168,000	323	364,000	700
$\:\stackrel{-}{\sigma\:}$	6062	4.6	27,288	34.5	12,117	5.2	55,922	40.9	132,955	254.1

**Fig. 5:**
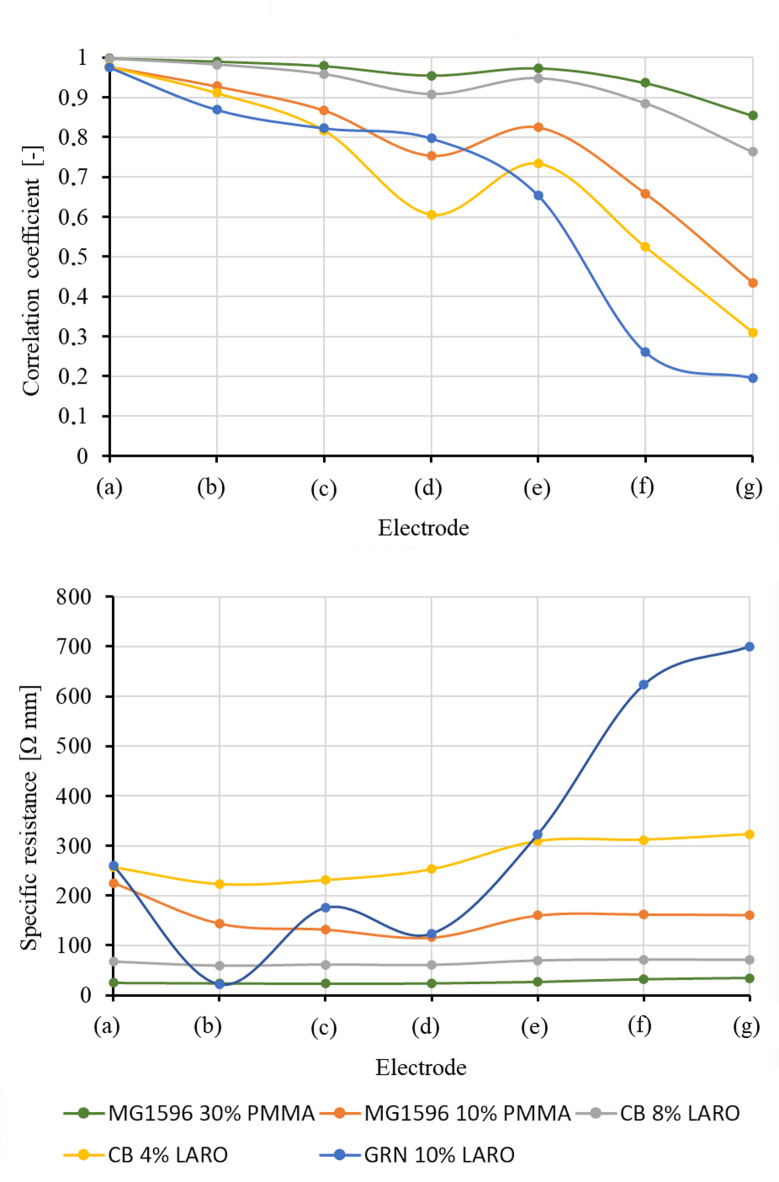
Electrical performance of the electrodes: correlation coefficient (upper graph), and specific resistance (lower graph).

We calculated the specific resistance for every electrode using the following equation:

1 $$\:{\rho\:}_{R}=\frac{R\cdot\:A}{L},$$

where $\:{\rho\:}_{R}$ – specific resistance, $\:L$ – length of electrode, $\:A$ – cross section of electrode. Figure 5 shows graphically the calculated correlation coefficients for every type of electrode (upper graph) and the values of $\:{\rho\:}_{R}$ (lower graph). The curves in Fig. 5 represent spline interpolations of the measurement points shown as dots. The measured values of the resistance $\:R$ and the specific resistance of the electrodes are shown in Table 3.

## Discussion

In our studies, we focused on electrical impulse conductors printed with three types of pastes, namely: fine-grained graphite MG1596 micro powder (10% and 30%), conductive carbon black CB (4% and 8%), and graphene GRN (10%). The goal of the research was to establish a correlation between the shape of the printed electrodes (length and width) and their ability to conduct electrical impulses. As carbon and carbon-derived materials exhibit excellent biocompatibility and low toxicity^27,28^, we studied two carbon allotropes, namely graphite, graphene, and black carbon.

Our results show that the electrical performance of electrodes depends on the composition of the printable paste and the shapes of the conductors. Figure 4; Table 2 demonstrate that the electrical efficiency of the widest electrodes (electrode (a)) is extremely high. However, the 25 mm wide electrodes cannot be used in nerve impulse conduction in vivo. Therefore, we studied electrodes with narrower widths (electrodes (b)÷(g)). Our experimental results indicate that as the electrodes’ width decreases, their electrical performance declines, resulting in higher resistance. Also, the longer electrodes, i.e. (e), (f), and (g), do not conduct electrical impulses efficiently. These observations are supported by the correlation coefficient values presented in Table 2. The highest correlation is observed for electrodes (a) and (b), while the lowest is for the 25 mm long electrode (d), and the 52 mm long electrodes (e)÷(g).

A higher amount of the carbon-driven constituent of the printable paste resulted in better conduction of electrical impulses. The higher the graphite or carbon black content the lower resistance (Table 3). This result is in good agreement with other studies^29^. An interesting observation is shown in Fig. 5, where the specific resistance (also referred to as resistivity) of the studied electrodes is depicted. It can be observed that MG1596 30% PMMA and CB 8% LARO produced the best results, as the resistance of the conductor layer was independent of the length or width of the printed electrodes. This is confirmed by the standard deviation of $\:R$, $\:{\rho\:}_{R}$, and the correlation coefficient, which is the lowest for those electrodes (Tables 2 and 3). For the other electrodes, noticeable changes in specific resistance are observed depending on electrode dimensions. This can be a result of the printing technique we used to manufacture the electrode. The screen printing method is a simple technique in which a paste is utilized to form the layer on the thin foil^30^. It has been demonstrated that screen printing enables the production of flexible, thin electrodes with a uniform microstructure and a uniform distribution of resistance across the layer surface^31^. The technique is also favorable for electrode fabrication due to its simplicity and cost-effectiveness^32^.

Analyzing Fig. 5; Tables 2 and 3 shows that specific resistance directly affects the correlation coefficient. Electrodes with higher resistivity exhibit a lower correlation coefficient, making them unsuitable for conducting electrical neural impulses.

Our studies were conducted in static conditions, which is a limitation of the research. The proposed carbon-based electrodes might change their properties and electrical performance in a potential in vivo application. The studies conducted on similar electrodes whose conductive material was also graphite, CB, or GRN showed low resistance change with bending cycles^33^. The results showed that materials of various compositions of the carbon types had decent adhesion to the substrate and were flexible, i.e. cyclic bending did not impact their properties. Other studies^34^, where the authors conducted tensile tests on electrodes containing graphite and CB in their conductive material, confirmed this observation. According to their results, the electrodes’ specific conductivity remained unchanged even under high strain (200%). Additionally, a polydimethylsiloxane composite containing exfoliated graphite demonstrates practically unchanged conductivity under cyclic tensile tests^35^. Another type of electrodes made of 3D interconnected graphene foam with many microchannels embedded within polydimethylsiloxane showed minimal alteration in relative resistance during cyclic bending at a 180^o^ angle^36^.

The electrical performance of the electrodes described in the present paper under dynamic conditions will be studied in the future. However, the results obtained by other authors allow us to expect that the properties of those electrodes will possibly remain unchanged. To ensure high electrical stability of the electrodes, we will embed the printed layer between the two foil films used in the present study.

In the present paper, the electrodes were tested by applying a single electrical signal. This is another limitation of our study. In the future, the electrical properties of electrodes under the influence of variable frequency impulses will be investigated.

## Conclusion

In the paper, we presented studies on the electrical performance of carbon-based electrodes concerning their application in neural impulse conduction for treating neural transmission disorders. We focused on electrodes produced using the screen printing technique. The printing paste was made of graphite, carbon black, and graphene.

Our studies show that graphite-based conductors containing 30% graphite (MG1596 30%) exhibit excellent electrical performance. Additionally, electrodes printed with a paste containing 8% of carbon black (CB 8% LARO) have outstanding electrical characteristics. The electrical conductance of the electrodes also depends on their dimensions. Our research proves that the wider the electrode, the higher the conductance. Surprisingly, graphene-based electrodes (GRN 10% LARO) showed relatively low electrical performance. It is hypothesized that increasing the amount of graphene in the printing paste could improve results.

There are many perspectives related to our study. First, various amounts of carbon constituents should be considered. Second, the type of binder can affect the electrical performance of the electrodes. Third, the influence of the electrodes’ deformation on impulse conductance is unknown. Those aspects will be addressed in future studies.

## Data Availability

The datasets used and/or analyzed during the current study are available from the corresponding author on reasonable request.
